# Myocardial late enhancement using dual-source CT: intraindividual comparison of single-energy shuttle and dual-energy acquisition

**DOI:** 10.1186/s13244-025-01944-4

**Published:** 2025-03-22

**Authors:** Takanori Kokawa, Kakuya Kitagawa, Satoshi Nakamura, Masafumi Takafuji, Takashi Oya, Hajime Sakuma

**Affiliations:** 1 Department of Radiology, Mie University Hospital, Tsu, Japan; 2 Department of Advanced Diagnostic Imaging, Mie University Graduate School of Medicine, Tsu, Japan; 3 Regional Co-creation Deployment Center, Mie Regional Plan Co-creation Organization, Mie University, Tsu, Japan; 4 Clinical Research Support Center, Mie University Hospital, Tsu, Japan; 5 Department of Radiology, Japanese Red Cross Ise Hospital, Ise, Japan

**Keywords:** Cardiac computed tomography, Dual-energy CT, Dual-source CT, Myocardial CT late enhancement, Extracellular volume

## Abstract

**Objectives:**

Myocardial computed tomography late enhancement (CT-LE) is a valuable modality used for the assessment of myocardial infarction and fibrosis and is effective in detecting latent cardiac amyloidosis. However, the optimal acquisition mode for CT-LE remains unknown. Here, we compared single-energy shuttle mode and DE mode for improving the quality of CT-LE imaging using dual-source CT.

**Methods:**

Fifteen patients with suspected or known ischemic heart disease underwent CT-LE imaging 5 min after coronary CT in both shuttle and dual-energy (DE) modes. In DE mode, virtual monoenergetic images at various keVs were reconstructed, and extracellular volume (ECV) was quantified using iodine-specific images. For shuttle mode, ECV was assessed by subtracting the volume from pre-contrast images from CT-LE after non-rigid registration.

**Results:**

In DE mode, signal-noise-to-ratio was the highest at 70 keV, but it was still lower than that in shuttle mode (*p* < 0.001). Contrast-noise-to-ratio was the highest on DE mode at 40 keV and was comparable with that in shuttle mode (*p* = 0.51). Interobserver agreement for infarct detection was higher in shuttle mode (kappa = 0.981) compared to DE mode (kappa = 0.808). Global ECV was comparable between shuttle and DE modes (*p* = 0.96). However, the coefficient of variation of segmental ECV was significantly lower in shuttle mode (*p* < 0.001).

**Conclusion:**

Shuttle mode CT-LE demonstrates superior image quality, better agreement in infarct detection, and ECV consistency in comparison to DE mode, suggesting its potential as the preferred approach for CT-LE imaging using dual-source CT despite limited z-axis coverage of 10.5 cm.

**Clinical relevance statement:**

CT late enhancement imaging in shuttle mode provides superior image quality and consistent extracellular volume measurements compared to dual-energy mode, highlighting its potential as the preferred acquisition method for CT late enhancement imaging in dual-source CT.

**Key Points:**

- Shuttle mode and dual-energy acquisition are compared for optimal myocardial CT-late enhancement (CT-LE) imaging.
- Shuttle mode can provide better image quality and more consistent extracellular volume measurements.
- Despite limited coverage, shuttle mode may be preferred for myocardial CT-LE imaging.

**Graphical Abstract:**

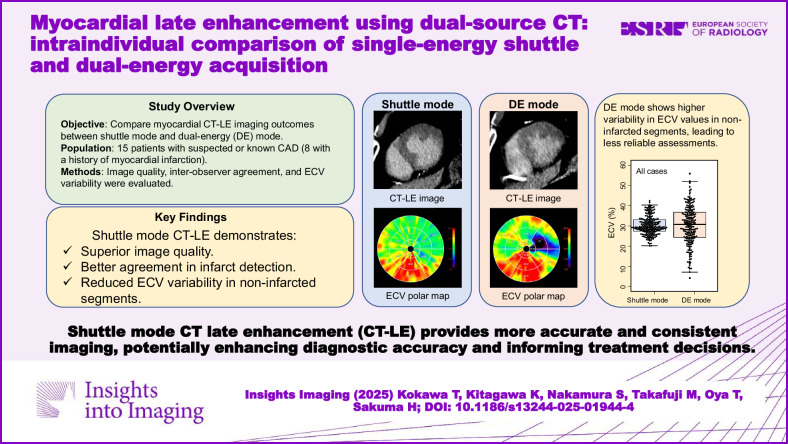

## Introduction

Myocardial computed tomography late enhancement (CT-LE) imaging has emerged as a valuable modality for the assessment of myocardial infarction (MI) and fibrosis [1, 2]. CT-LE for MI has comparable diagnostic performance to magnetic resonance imaging (MRI), and it is also useful for risk stratification [3, 4]. Furthermore, CT-LE is particularly effective in detecting latent cardiac amyloidosis, especially in patients with aortic stenosis who are candidates for transcatheter aortic valve replacement procedures [5]. However, despite good agreement between CT and MRI, CT-LE often exhibits relatively poorer contrast-to-noise ratio (CNR) compared with late gadolinium enhancement (LGE)-MRI [6]. Additionally, various artifacts may degrade image quality, and the sensitivity for detecting lesions, such as infarction and fibrosis, may be lower compared with that of LGE-MRI [7]. Accordingly, several strategies have been explored to address these limitations and enhance the quality of CT-LE imaging [8–11]. Dual-energy (DE) imaging is a promising approach, which can enhance the contrast between the myocardial scar and the normal myocardium by utilizing low keV virtual monoenergetic images (VMIs) or iodine-specific images [12–14]. Moreover, it allows for the direct calculation of extracellular volume (ECV) without the need for pre-contrast images [15].

There have been innovations in image reconstruction methods in single-energy imaging. Specifically for dual-source CT, the hybrid reconstruction method effectively combines low spatial frequency data from full-scan reconstruction with high spatial frequency data from partial-scan reconstruction, thereby minimizing myocardial CT number variations caused by the X-ray source position while maintaining high temporal resolution and reducing motion artifacts [16]. Despite these advances, the optimal acquisition mode for CT-LE remains unknown. Particularly, there have been no direct comparisons between the two approaches for dual-source CT (i.e., DE and shuttle modes).

Therefore, our study aimed to compare these imaging approaches and evaluate their effects on image quality, radiation dose, and ECV quantification.

## Materials and methods

### Ethics and study population

This study was a substudy of a larger prospective research project investigating comprehensive cardiac CT protocols, including stress dynamic CT perfusion and delayed enhancement CT approved by our institutional review board and was conducted according to the tenets of the Declaration of Helsinki. Written informed consent for participation in the study was obtained from all patients.

A total of 15 consecutive patients scheduled to undergo a comprehensive cardiac CT examination, including stress dynamic CT perfusion and CT-LE, were prospectively enrolled from July to September 2018. Inclusion criteria were (1) patients with suspected or known coronary artery disease, and (2) age 45–85 years. Exclusion criteria were (1) patients with impaired breath holding and (2) patients with renal failure.

### CT data acquisition and reconstruction

**Fig. 1:**
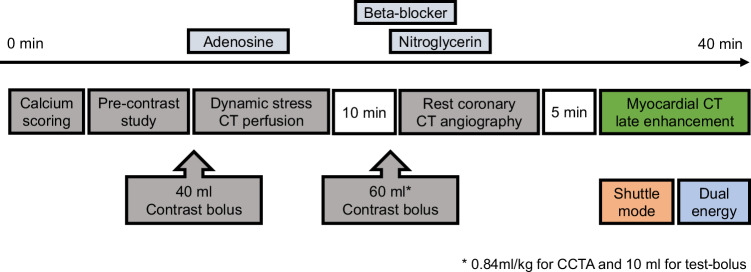
Image acquisition timeline of cardiac CT examination. Calcium scoring, pre-contrast CT, dynamic stress CT perfusion, resting coronary CT angiography, and myocardial CT late enhancement are performed. CT late enhancement images are acquired with both shuttle mode and dual-energy mode

All cardiac CT examinations were performed using a third-generation, dual-source CT system (SOMATOM Force; Siemens Healthcare, Forchheim, Germany). CT-LE was performed as part of a comprehensive study that included pre-contrast CT, stress dynamic myocardial perfusion CT, and rest coronary CT angiography [8, 17, 18] (Fig. 1). Scout images of the entire chest were obtained, and then calcium scoring was performed. Subsequently, pre-contrast CT images for calculating the ECV were acquired at the end-systolic phase using a method for acquiring CT-LE, as explained later. Adenosine stress dynamic myocardial perfusion CT of 30 s at two alternating table positions was performed with a bolus injection of 40 mL with an iodine concentration of 370 mgI/mL (Iopamiron 370 syringe 100 mL; Bayer Schering Pharma, Berlin, Germany). Prospectively electrocardiography (ECG)-triggered coronary CT angiography was acquired 10 min after stress perfusion CT with 60 mL of contrast medium using the following scan parameters: tube voltage, 80 kVp; mAs per rotation, 580; and rotation time, 250 ms [8, 19]. Heart rate was controlled before coronary CT angiography with intravenous injection of landiolol hydrochloride (Corebeta, Ono Pharmaceutical Co., Ltd, Osaka, Japan).

At 5 min after coronary CT, myocardial CT-LE images were acquired in both shuttle and DE mode without additional contrast medium administration (100 mL iodine contrast medium per patient) [19]. The shuttle mode CT-LE protocol has been described in detail elsewhere [8, 20] and included as a Supplementary Method, but briefly, was acquired using a prospective ECG-triggered axial scan at two alternating table positions with a z-axis coverage of 10.5 mm during end-systole (250 ms after R wave) with 96 × 0.6 mm collimation and 80 kVp tube voltage. The tube current was determined by an automatic exposure control system with quality reference mAs settings at 580 mAs/80 kVp. Overall, three image stacks were acquired during 1 breath hold and reconstructed with 1-mm slice thickness and 1-mm increment with a Qr36d kernel. The three image stacks (each containing 104 slices) were averaged to a single final image stack using non-rigid registration processed with volume perfusion software (Syngo via VA30; Siemens Healthcare) for noise reduction. Pre-contrast CT was performed using the same acquisition protocol as the late-phase CT [21, 22].

DE mode CT-LE was performed immediately after the shuttle mode, within 1 min without any delay. DE mode was acquired by using retrospective ECG-gated spiral acquisition with ECG pulsing window in 20–40% of the R-R interval, 96 × 0.6 mm collimation, 90 kV with 165 mAs, and Sn150 kV with 127 mAs. The rotation time was 250 ms [12]. Scanning range and field of view were adjusted according to heart size. Axial images were reconstructed at 250 ms after the R wave using a 0.75-mm section thickness, 0.75-mm increment interval, and a medium-smooth convolution kernel (Qr40d). Half-scan and full-scan reconstructions could be selected in the DE mode, but the current study used full-scan reconstruction because it provided better image quality (Supplementary Fig. 3). VMIs at 40, 60, 70, and 80 keV; a mono-plus at 40 keV; and iodine-specific images were generated using a commercially available workstation (Syngo via 30A; Siemens Healthcare).

For each patient, the dose-length product (DLP) was recorded using an automatically generated patient protocol.

### Image analysis

To assess the image quality of CT-LE, a region of interest (ROI) was positioned in the left ventricular (LV) myocardium of the septum without late enhancement, infarcted myocardium, or LV cavity (LVC) on axial CT-LE, with a 6-mm slice thickness. In 17 myocardial segments defined by the American Heart Association, excluding the apex, the presence or absence of infarction was independently evaluated by two observers for all shuttle mode and DE mode reconstructions. Any discrepancies between the observers were resolved through consensus. The signal-to-noise ratio (SNR) of the LV myocardium (SNR_myo_), LVC (SNR_LVC_), and CNR were determined as follows:

$${{SNR}}_{{myo}}={mean\; HU}({septal\; myocardium})/{SD}({septal\; myocardium})$$

$${{SNR}}_{{LVC}}={mean\; HU}({LV\; cavity})/{SD}({LV\; cavity})$$

$${CNR}=\frac{\left[{HU}\left({infarcted\; myocardium}\right)-{HU}\left({septal\; myocardium}\right)\right]}{{SD}({septal\; myocardium})}$$

**Fig. 2:**
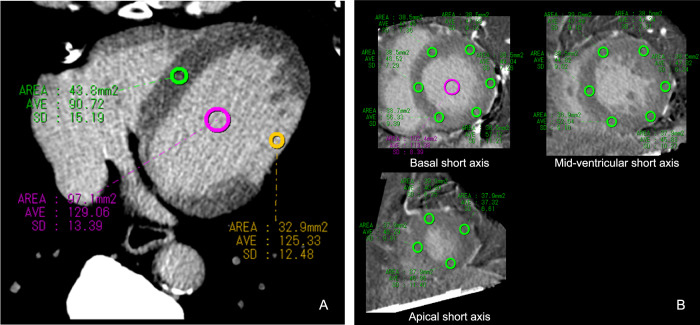
Quantitative evaluation of image quality involves placing circular ROIs in the MI segment (yellow), the non-MI segment (green), and the LVC (pink) on the axial plane to measure the SNR and the CNR (**A**). To evaluate global and segmental ECV, a circular ROI is positioned in each myocardial segment and LVC across three short-axis planes: basal, mid-ventricular, and apical subtraction images (shuttle mode) or iodine-specific images (dual-energy mode) (**B**). For clarity, **A** and **B** utilize different cases, both captured in shuttle mode. **A** features a myocardial CT late enhancement image, whereas **B** presents subtraction images. ROI, region of interest; MI, myocardial infarction; SNR, signal-to-noise ratio; CNR, contrast-to-noise ratio; LVC, left ventricular cavity; ECV, extracellular volume

In shuttle mode, ECV is calculated with the following equation: ECV = (ΔHUmyo/ΔHUblood) × (1 − Hct), where ΔHUmyo and ΔHUblood represent changes in the attenuation of the myocardium and LVC in Hounsfield units (HU), respectively, before and after contrast administration. To obtain ΔHU, pre-contrast CT images were subtracted from CT-LE images on a workstation (Ziosoft Inc., Tokyo, Japan) using an automatic non-rigid image registration function [18]. Subtraction images were displayed in the cardiac short axis, and circular ROI were manually placed in 17 American Heart Association myocardial segments except for the apex to obtain the ΔHU in each segment. The ΔHUblood was measured by placing a circular 1.0-cm^2^ ROI in the LVC. In DE mode, ECV was calculated from iodine-specific images with the following equation: ECV = (iodine density in the myocardium/iodine density in the LVC) × (1 − Hct), by placing the same ROI used for the analysis of subtraction image (Fig. 2). The global ECV was calculated as the mean ECV of the 16 segments after excluding segments with MI on the CT-LE images. The regional variation in ECV within a patient was assessed by evaluating the coefficient of variation (CoV) of the ECV in segments without late enhancement, which was defined as 100 × (standard deviation [SD] of segmental ECV) / (global ECV).

### Statistical analysis

Continuous variables are expressed as means and SDs and are compared using the paired Student’s *t*-test after examining the normality of data distribution. Meanwhile, categorical variables are expressed as proportions, and the differences in proportions are assessed using the chi-square test. Interobserver agreement was evaluated using Cohen’s kappa (κ) statistic to account for agreement beyond chance. Kappa values were interpreted as follows: κ < 0.20, slight agreement; κ = 0.21–0.40, fair agreement; κ = 0.41–0.60, moderate agreement; κ = 0.61–0.80, substantial agreement; and κ = 0.81–1.00, almost perfect agreement. The 95% confidence intervals (CIs) for kappa were calculated. Additionally, the percentage of observed agreement was reported to provide a direct comparison between observers. All statistical analyses were performed using the GraphPad PRISM 6 software (GraphPad Software, Inc., LaJolla, CA). Significance was set at *p* < 0.05, and the Bonferroni correction was applied where appropriate.

## Results

### Baseline patient characteristics

The final cohort comprised 12 men and 3 women. Table 1 shows the baseline patient characteristics. The mean (SD) age was 69.3 ± 8.3 years, and the mean (SD) body mass index was 22.3 ± 1.6 kg/m^2^. The mean administered iodine dose was 599.5 ± 78.4 mgI/kg. The mean (SD) heart rate during shuttle mode and DE mode acquisition was 65.9 ± 8.4 and 64.9 ± 8.5 bpm, respectively. Patients with atrial fibrillation were not included in this study population.

**Table 1:** Baseline patient characteristics

Characteristic	
Age (years)	69.3 ± 8.3
Male sex, *n* (%)	12 (80.0)
Body mass index (kg/m^2^)	22.3 ± 1.6
Hematocrit (%)	36.5 ± 4.9
Risk factor, *n* (%)	
Hypertension	13 (86.7)
Dyslipidemia	8 (53.3)
Diabetes	7 (46.7)
Smoking	3 (20.0)
Family history	3 (20.0)
History of coronary artery disease, *n* (%)	
History of myocardial infarction	8 (53.3)
Prior coronary artery bypass grafting	2 (13.3)
Prior percutaneous coronary intervention	7 (47.7)

Data are presented as the mean ± standard deviation

### Detection of MI, SNR and CNR

Among the 15 cases, 8 had a history of MI, and both DE and shuttle mode CT-LE successfully detected infarction in all 8 cases, although sensitivity varied among DE reconstructions (Supplementary Table). The results of the segment-based analysis are summarized in Table 2. Using the shuttle mode, an almost perfect agreement was achieved, with a κ value of 0.981. In contrast, agreement for dual-energy reconstruction ranged from moderate to substantial (κ = 0.581–0.808), primarily due to the frequent under-detection of MI. Based on these findings, a consensus between the two readers was reached regarding the location of infarction. A total of 30 MI segments and 210 non-MI segments were identified.

**Table 2:** Interobserver agreements for MI segment detection

	Dual-energy mode										Shuttle mode	
	Mono-plus 40 KeV		40 KeV		60 KeV		70 KeV		80 KeV			
Observer	A	B	A	B	A	B	A	B	A	B	A	B
Non-MI segments	214	219	220	229	219	221	211	219	221	219	210	211
MI segments	26	21	20	11	21	19	29	24	29	24	30	29
κ value	0.808		0.646		0.581		0.698		0.698		0.981	
Agreement	96.7% (232/240)		95.8% (230/240)		93.8% (225/240)		94.2% (226/240)		94.2% (226/240)		99.6% (239/240)	

Values are numbers of segments

κ value between each keV of dual-energy mode and shuttle mode (inter-test agreement)

*MI* myocardial infarction, *CT-LE* CT late enhancement

**Fig. 3:**
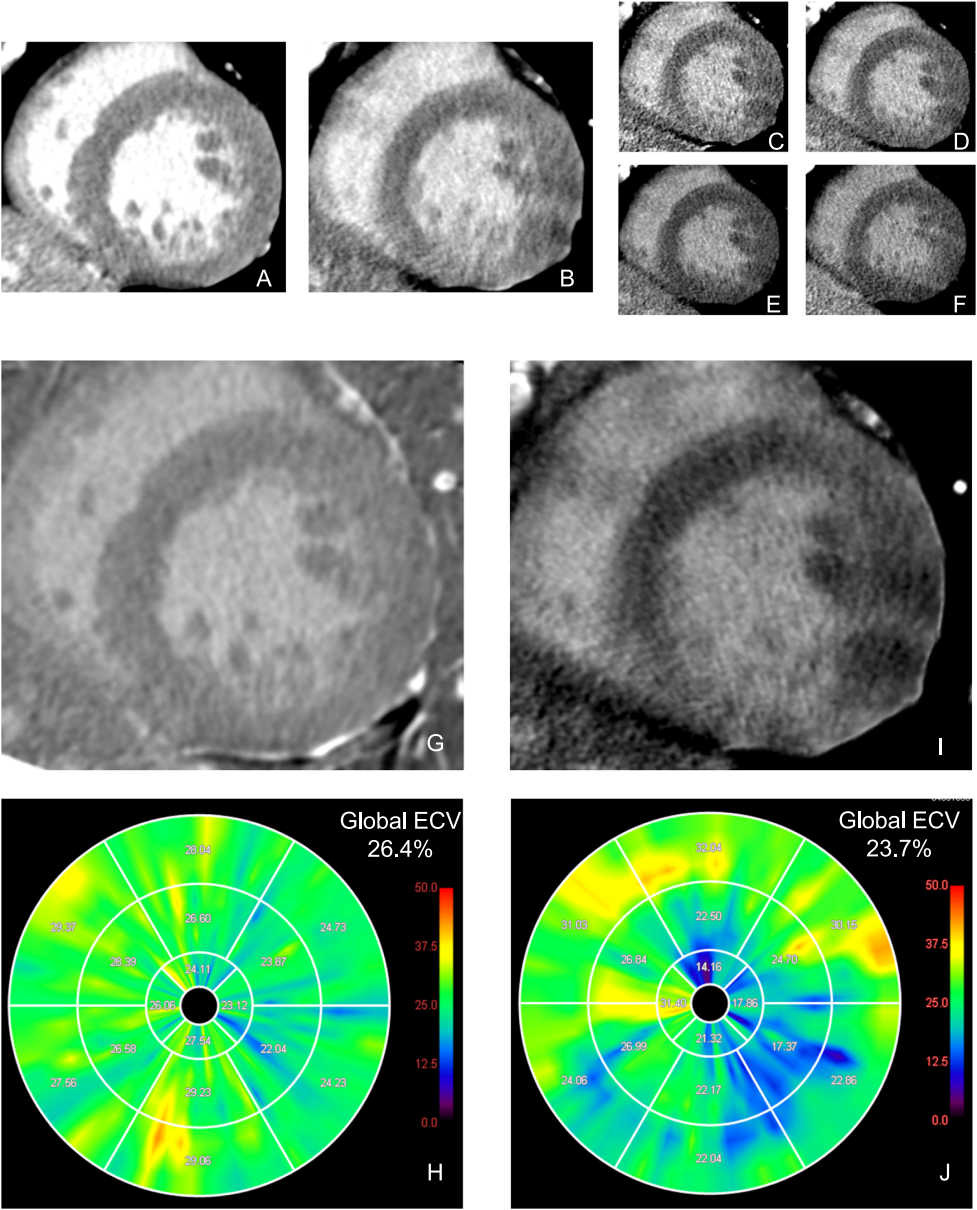
CT-LE in a 75-year-old man without history of myocardial infarction undergoing percutaneous coronary intervention for angina pectoris. CT-LE images include shuttle mode (**A**) and DE mode images: mono-plus 40 keV (**B**), 40 keV (**C**), 60 keV (**D**), 70 keV (**E**), 80 keV (**F**). A short-axis subtraction image (**G**) and a polar map of ECV (**H**) derived from subtraction images and an iodine-specific image (**I**) and a polar map of ECV (**J**) derived from iodine-specific images are also presented. Streak artifacts are more pronounced in DE mode than in shuttle mode, resulting in a larger variation in segmental ECV. CT-LE, CT late enhancement; DE, dual-energy; ECV, extracellular volume

**Fig. 4:**
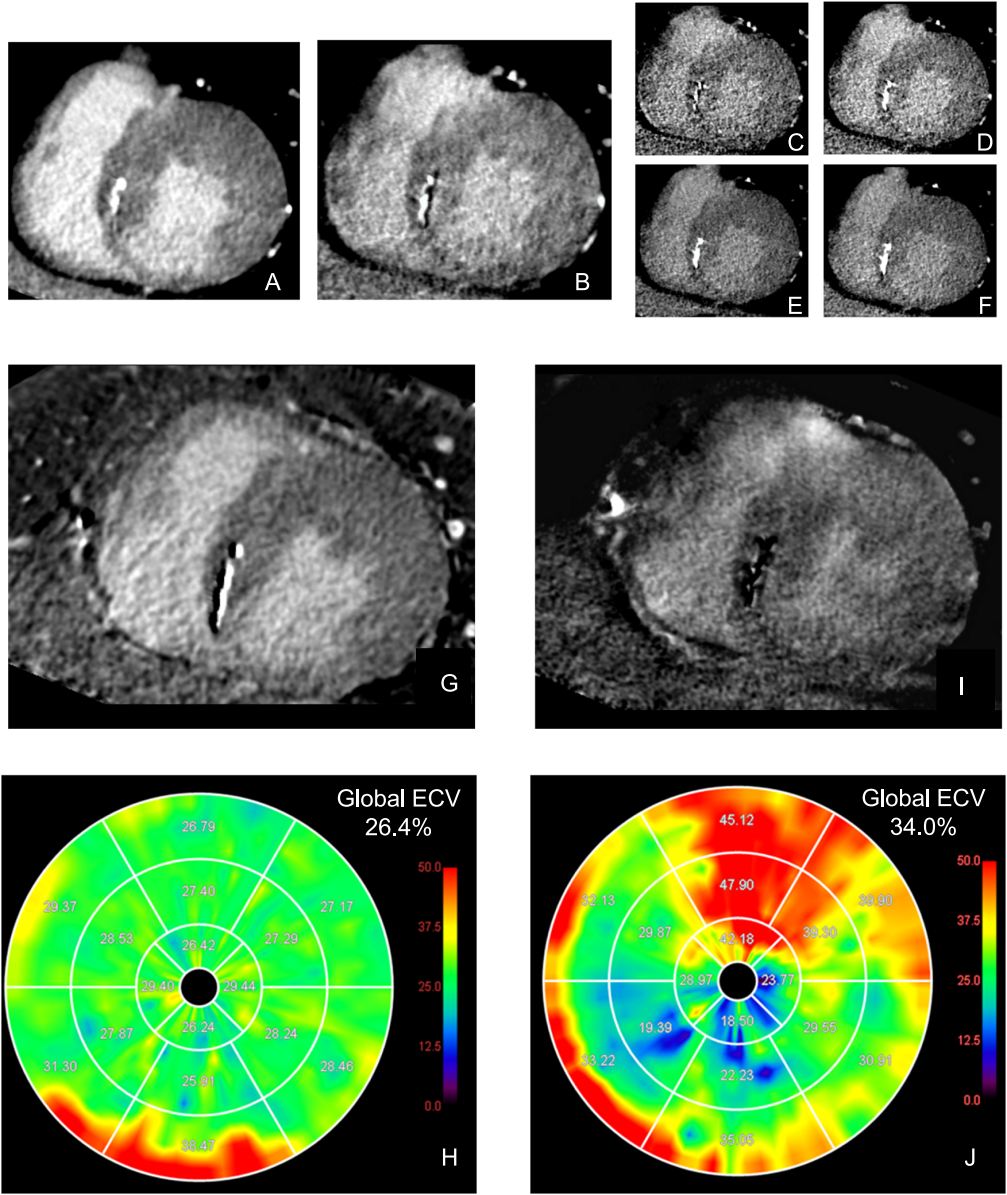
CT-LE in a 57-year-old man with severe inferior wall hypokinesis on transthoracic echocardiography and chronic total occlusion of the proximal right coronary artery on coronary CT angiography. CT-LE images include shuttle mode (**A**) and DE mode images: mono-plus 40 keV (**B**), 40 keV (**C**), 60 keV (**D**), 70 keV (**E**), 80 keV (**F**). A short-axis subtraction image (**G**) and a polar map of ECV (**H**) derived from subtraction images and an iodine-specific image (**I**) and a polar map of ECV (**J**) derived from iodine-specific images are also presented. Shuttle mode images (**A**) clearly show subendocardial infarction in the basal inferior wall, whereas DE mode images (**B**–**F**) do not depict this finding as distinctly. Shuttle mode reveals an increase in ECV in the basal inferior segments reflecting late enhancement (**G**, **H**). In contrast, DE mode shows no correlation; instead, an increase in ECV is observed in the anterior wall, unrelated to the infarct. Note that the linear high attenuation in the interventricular septum likely represents septal branch calcification rather than a region of late enhancement. CT-LE, CT late enhancement; DE, dual-energy; ECV, extracellular volume

The SNR and CNR are summarized in Table 3. In the shuttle mode, the SNRs in the myocardium and LVC were 12.9 ± 3.0 and 19.2 ± 2.1, respectively. The SNR in the myocardium and LVC were the highest in the DE mode at 70 keV, with values of 8.6 ± 1.6 and 11.8 ± 2.5, respectively. Meanwhile, the SNR was significantly higher in shuttle mode than in DE mode at 70 keV (*p* < 0.001 for both the myocardium and LVC). The CNR was calculated for eight patients in whom MI was detected. The CNR in shuttle mode was 4.3 ± 1.0. In DE mode, the highest CNR was 3.8 ± 1.7 with mono-plus 40 keV, which was lower than that in shuttle mode; however, this difference was not statistically significant (*p* = 0.51). Representative patients undergoing CT-LE using the shuttle and DE modes are presented in Figs. 3 and 4, respectively.

**Table 3:** Pairwise comparison of signal- and contrast-to-noise ratio between shuttle mode scan and dual-energy mode scan

CT examination	SNR_myo_	*p-*values	SNR_LVC_	*p-*values	CNR	*p-*values
Shuttle mode	12.9 ± 3.0		19.2 ± 2.1		4.3 ± 1.0	
Dual-energy mode						
Mono-plus 40 Kev	6.4 ± 1.2	< 0.001	11.2 ± 2.3	< 0.001	3.8 ± 1.7	0.51
40 KeV	3.2 ± 0.6	< 0.001	5.0 ± 1.8	< 0.001	1.9 ± 0.9	< 0.001
60 KeV	6.3 ± 1.2	< 0.001	9.4 ± 1.9	< 0.001	2.4 ± 0.9	< 0.001
70 KeV	8.6 ± 1.6	< 0.001	11.8 ± 2.5	< 0.001	2.5 ± 0.6	0.002
80 KeV	8.3 ± 1.6	< 0.001	10.7 ± 2.1	< 0.001	1.8 ± 0.3	< 0.001

*SNR*_*myo*_ signal-to-noise ratio of the left ventricular myocardium, *SNR*_*LVC*_ signal-to-noise ratio of the left ventricular cavity, *CNR* contrast-to-noise ratio

### Global ECV and variation of segmental ECV

**Fig. 5:**
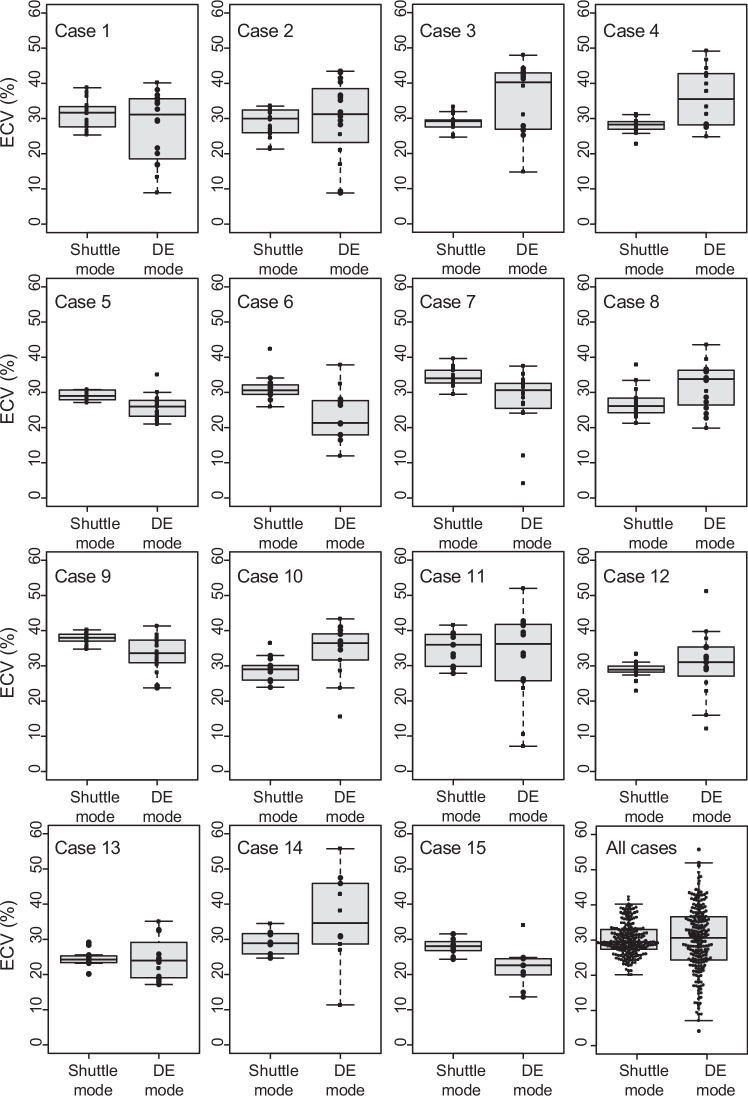
Box-and-whisker plot of ECV of non-MI segments derived from shuttle mode and DE mode in all 15 patients. The results clearly demonstrate that shuttle mode exhibits less variability in ECV compared to DE mode, with no segments showing ECV below 20%. MI, myocardial infarction; ECV, extracellular volume; DE, dual-energy

Global ECV from the shuttle mode (subtraction images) and DE mode (iodine-specific images) was comparable (30.0 ± 3.3% vs 30.1 ± 4.5%, *p* = 0.96). However, the ECV in segments without MI (*n* = 210) varied differently between the shuttle mode and DE mode (20.2–42.6% vs 4.2–55.8%). Further, the patient-based CoV of segmental ECV, calculated from segments without MI, was significantly lower in shuttle mode than in DE mode (9.8 ± 3.2% vs 26.5 ± 7.3%, *p* < 0.001), indicating less variation in shuttle mode. None of the segments had excessively low ECV (< 20%) in shuttle mode, whereas 28 segments (13.3%) had excessively low ECV in DE mode. Moreover, only 3 segments (1.4%) exhibited excessively high ECV (> 40%) in shuttle mode, in contrast to 32 segments (15.2%) in DE mode (Fig. 5).

### Radiation dose

The DLP of CT-LE on shuttle mode was significantly lower than that of CT-LE on DE mode (149.1 ± 16.7 mGy∙cm vs 224.7 ± 43.6 mGy∙cm, *p* < 0.001). Even when including the DLP of pre-contrast CT scan, the DLP was lower for CT-LE on shuttle mode than for CT-LE on DE mode (196.8 ± 21.6 mGy∙cm vs 224.7 ± 43.6 mGy∙cm, *p* = 0.044). The mean DLP for the entire cardiac examination was 822.0 ± 132.0 mGy･cm (Table 4).

**Table 4:** Dose-length product (mGy·cm)

Calcium scoring	14.2 ± 6.3
Pre-contrast CT	47.8 ± 5.5
Stress dynamic CT perfusion	224.8 ± 44.0
Coronary CT angiography	168.4 ± 65.8
CT late enhancement	
Shuttle mode	149.1 ± 16.7
Dual-energy mode	224.7 ± 43.6
Total	822.0 ± 132.0

## Discussion

In this intraindividual comparison of two approaches for CT-LE imaging and ECV assessment, shuttle mode demonstrated higher SNR and higher interobserver agreement in detecting MI segments compared with DE mode. Although CNR and global ECV were comparable between the two approaches, the regional variation in ECV in segments without late enhancement within a patient was smaller with shuttle mode, which may place shuttle mode a preferred approach.

The cardiac shuttle mode was originally developed to enable dynamic myocardial CT perfusion imaging in dual-source CT. This technique is specifically designed for dual-source CT systems and cannot be implemented on single-source CT scanners at present. This mode integrates components of full-scan and half-scan reconstruction to optimize image quality and diagnostic accuracy. In full-scan reconstruction, a 360° dataset provides detailed contrast information but sacrifices temporal resolution. In contrast, half-scan reconstruction uses a 180° dataset to enhance temporal resolution but introduces increased noise and artifacts caused by variations in X-ray source position (partial-scan artifacts). Data acquired with cardiac　shuttle mode should be reconstructed with a special reconstruction algorithm called targeted spatial frequency filtration that combines low-frequency components from full-scan reconstruction, which captures contrast information, with high-frequency components from half-scan reconstruction, which convey anatomical details. This integration reduces noise, minimizes partial-scan artifacts, and ensures consistent CT numbers. Phantom and animal studies have demonstrated the effectiveness of this technique in achieving accurate and reproducible CT values of the LV myocardium [16]. Kurobe et al were among the first researchers to apply the shuttle mode to CT-LE imaging. They demonstrated that this technique achieves higher SNR and CNR, along with improved interobserver reproducibility of infarct sizing and better agreement with LGE-MRI compared to conventional prospective half-scan technique [8]. One limitation of shuttle mode is its limited z-axis coverage. In a study on patients with ischemic heart disease, incomplete LV coverage was observed in 56% of cases with second-generation dual-source CT (7 cm coverage) and in 1.9% with third-generation CT (10.5 cm coverage) [20]. In our study using third-generation CT, complete LV coverage was achieved in all cases. However, in patients with left ventricular dilatation, regions such as the basal anterior wall and apical inferior wall may extend beyond the imaging range.

DE CT is a relatively recent advancement that utilizes two different energy spectra of X-rays to acquire attenuation data of the body. This enables DE CT to provide various image sets, including iodine-specific images, virtual non-contrast images, effective atomic number images, and VMIs. Furthermore, DE CT can effectively correct beam-hardening artifacts originating from the chest wall through VMI, as beam hardening mainly results from the polychromatic nature of X-rays utilized in clinical CT scanners [23]. VMI at low keV enhances CT-LE visualization [12, 13, 24]. Iodine-specific images allow direct calculation of ECV without subtraction of pre-contrast CT from CT-LE, potentially allowing radiation reduction and elimination of misregistration [7]. However, different vendors have developed distinct hardware and software approaches for generating DE datasets, each with its own advantages and disadvantages [25]. Dual-source CT DE scans exhibited a high concordance with MRI-derived ECV and demonstrated strong agreement with MRI in detecting MI and fibrosis [12, 26, 27]. Nevertheless, in cardiac DE scanning with dual-source CT, the temporal resolution is comparable to that of single-source CT, which is less than ideal for capturing the dynamic nature of cardiac structures. Additionally, current software does not support prospective DE scans, which restrict flexibility in radiation dose optimization. These specific limitations of dual-source CT DE scans may help explain the image quality observed in this study. First, the comparable temporal resolution to single-source CT likely contributes to the higher variation in ECV measurements. Second, because only retrospective scans are feasible, significant radiation exposure occurs across the entire cardiac cycle, extending beyond the systolic phase used for late enhancement imaging in our study. This results in unnecessary radiation exposure throughout the entire cardiac cycle and may dilute dose concentration during the target phase when total radiation is kept comparable to that of a prospective scan. Early reports on CT-LE using dual-source photon-counting detector CT, which successfully integrates high temporal resolution with DE processing, have shown promising results for ECV measurement [28, 29]. Further studies are needed to determine whether hybrid reconstruction, such as that used in this study, is also useful for dual-source photon-counting detector CT.

This study is limited by its small sample size and single-center design. The present comparison between single- and DE systems is applicable only to third-generation dual-source CT systems and not to other CT systems. As shuttle mode is unique to dual-source CT systems, results may differ when using other DE approaches, such as dual-layer-detectors or rapid kV switching methods on single-source CT systems. For example, a study utilizing dual-layer CT demonstrated that the DE method achieved superior ECV measurement accuracy compared to the single-energy methods [30]. This highlights the potential variability in performance across different CT platforms and underscores the need for further studies to validate these findings in other system configurations. We acknowledge that direct comparisons with MRI and ex vivo studies were not conducted in this study, in this regard, it primarily serves as a proof of concept. However, prior researchers [8, 22] have demonstrated high concordance between shuttle-mode CT and MRI-derived parameters, supporting the reliability of the shuttle technique. In addition, the shuttle mode is limited by its short z-axis coverage of 10.5 cm. Future research should involve larger, multi-center studies with broader CT system comparisons, validation using LGE-MRI and MRI-derived ECV, and the development of solutions to overcome shuttle mode’s z-axis coverage limitation. Lastly, we administered 100 mL of contrast medium to all patients, in accordance with the specifications of the syringe preparation used at our institution. The administered iodine dose (mean: 599.5 ± 78.4 mgI/kg) falls within the recommended range of 550–600 mgI/kg for CT-LE [7]. However, considering that Lee et al reported administering 666 mgI/kg in their CT-LE studies using the dual-source DE method [26, 27], higher contrast doses might have yielded even better results for the DE method in our study.

## Conclusion

In myocardial late enhancement imaging with dual-source CT, shuttle mode significantly enhances SNR and CNR compared to DE mode, without increasing radiation dose. This enhancement leads to higher interobserver agreement in the detection of MI segments. Additionally, it provides more consistent ECV measurements across non-MI segments. These findings suggest that shuttle mode may be the preferred method for CT-LE imaging, providing superior image quality despite its limited z-axis coverage of 10.5 cm.

## Supplementary information

ELECTRONIC SUPPLEMENTARY MATERIAL

## Data Availability

Data will be made available on reasonable request.

## Abbreviations

CNR: Contrast-to-noise ratio
CoV: Coefficient of variation
CT-LE: Computed tomography late enhancement
DE: Dual energy
DLP: Dose-length product
ECV: Extracellular volume
LGE: Late gadolinium enhancement
LVC: Left ventricular cavity
MI: Myocardial infarction
SNR: Signal-to-noise ratio
