# Supplementary material for: Myocardial late enhancement using dual-source CT: intraindividual comparison of single-energy shuttle and dual-energy acquisition
Source: Insights Imaging. 2025 Mar 22;16:64. doi: 10.1186/s13244-025-01944-4 (PMC11929652; doi:10.1186/s13244-025-01944-4)
Supplement: Supplementary file 1 — ELECTRONIC SUPPLEMENTARY MATERIAL [file 13244_2025_1944_MOESM1_ESM.pdf]

Myocardial late enhancement using dual-source CT: Intraindividual comparison of single-energy shuttle and dual-energy acquisition
ELECTRONIC SUPPLEMENTARY MATERIAL

Supplementary Method

The acquisition scheme for dynamic CT perfusion imaging, available on dual-source CT systems (Somatom Definition Flash, Somatom Force, Somatom Drive, Siemens Healthcare), serves as the foundation for “shuttle mode” CT late enhancement (CT-LE) imaging. Below is a detailed explanation of its application:

Myocardial Dynamic CT Perfusion Data Acquisition

Myocardial dynamic CT perfusion imaging acquires data using two alternating table positions (Position 1 and Position 2 in Figure 1) in an ECG-triggered mode during the end-systolic phase. This setup:

- Utilizes table movement (“shuttle mode”) with an overlap of 10% between imaging ranges.
- Achieves a z-axis coverage of **105 mm (Force) or 73 mm (Definition Flash, Drive)** (sufficient to image the entire myocardium in its end-systolic state).
- Captures 8–14 dynamic phases over a 30-second period to monitor the first pass of the contrast medium through the heart, providing robust data for quantitative myocardial perfusion analysis. (Bamberg F, Eur Radiol 2010)

Figure 1

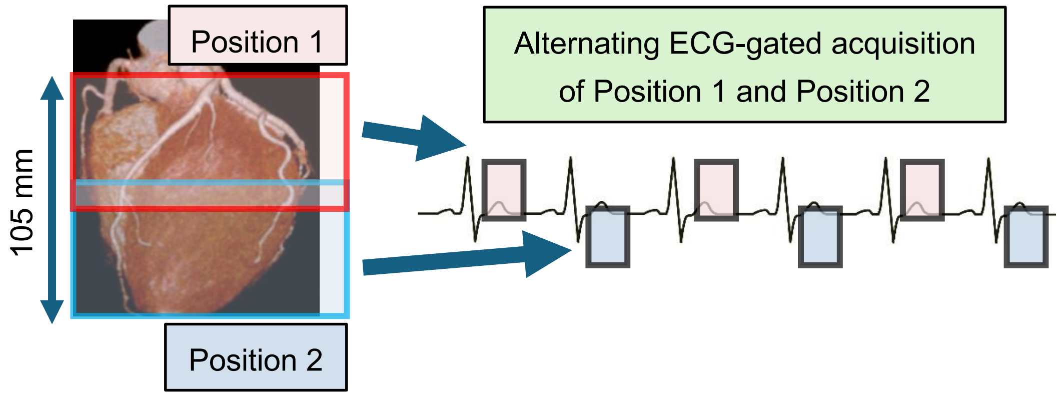

Modifications for CT-LE Imaging

For CT-LE imaging, the dynamic CT perfusion methodology is adapted with the following changes to meet the requirements of late enhancement imaging:

1. **Reduction in Dynamic Phases**:
   - Instead of capturing 8–14 phases, CT-LE acquires only **3 dynamic phases (or "stacks")**, reflecting the reduced temporal demand of late enhancement imaging compared to perfusion imaging.
2. **Noise Reduction through Averaging**:
   - After acquisition, the 3 nearly identical stacks are averaged after non-rigid registration, effectively reducing image noise and enhancing clarity (Figure 2). This step is particularly crucial for delineating late enhancement with high accuracy. (Kurobe Y, JCCT 2014)

Figure 2

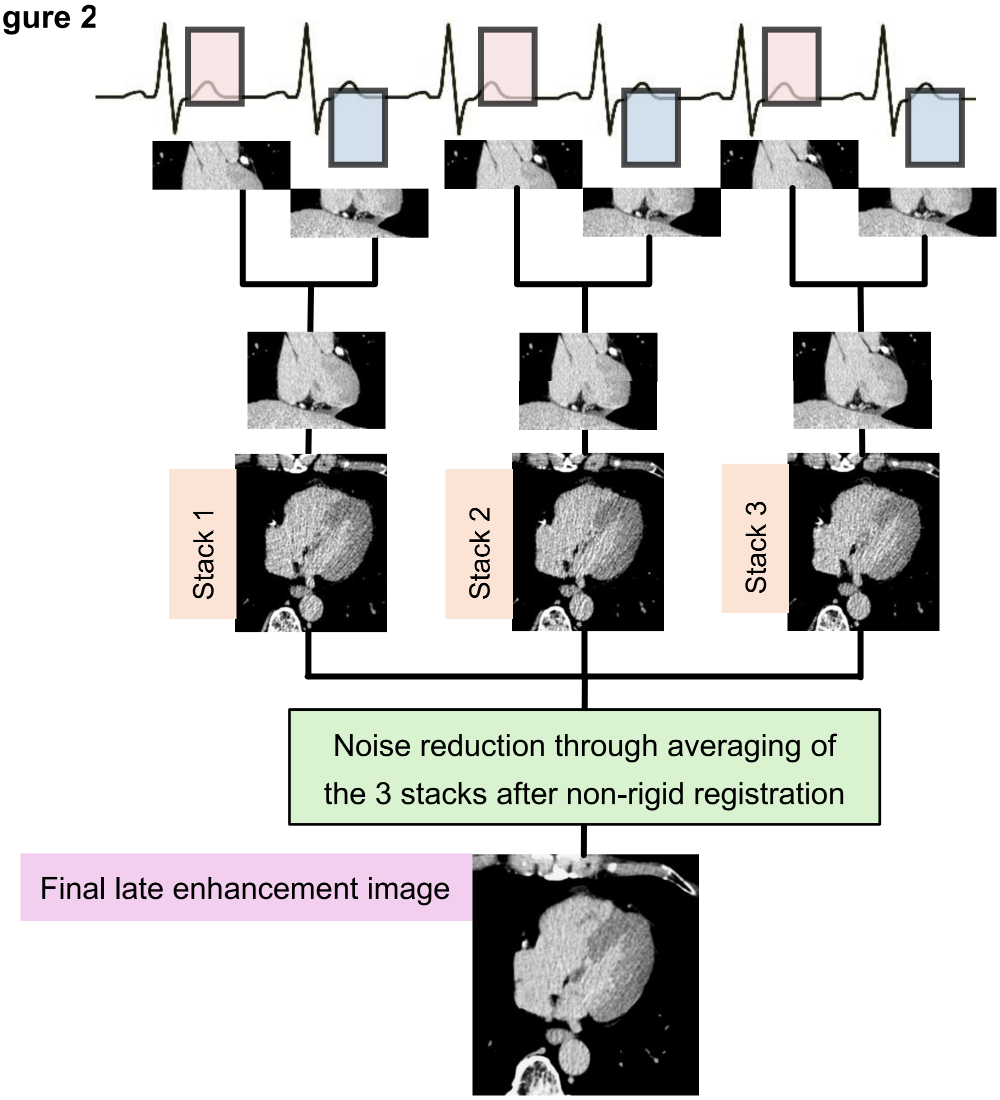

Insights Imaging (2025) Kokawa T, Kitagawa K, Nakamura S, Takafuji M, Oya T, Sakuma H.

Dedicated Reconstruction Method for Dynamic Imaging

Myocardial dynamic CT perfusion imaging relies on a specialized reconstruction method to ensure high temporal resolution and CT value stability, which is also utilized in CT-LE imaging:

1. **Partial and Full Image Reconstruction**:
   - Partial image reconstruction used in continuously rotating CT devices achieves high temporal resolution but introduces variability in CT values due to the lack of 360° symmetry. These artefacts are comparable in magnitude to the expected myocardial enhancement, leading to inconsistent HU values (McCollough CH, Med Phys 2008).
   - The hybrid reconstruction approach combines:
     - **Low-frequency components** (contrast information) from a **360° reconstruction** (temporal resolution: 188 ms for Force, 210 ms for Definition Flash, Drive).
     - **High-frequency components** (anatomical details) from a **partial (180°) reconstruction** (temporal resolution: 66 ms for Force, 75 ms for Definition Flash, Drive.
   - This combining ensures images with high temporal resolution and stable CT values. (Ramires-Giraldo, JC, Med Phys 2011)
2. **Limitations in Noise Reduction**:
   - The reconstruction method is based on filtered back projection, and iterative reconstruction methods are not available for noise reduction.
   - To minimize noise, **3 dynamic phases** are acquired and averaged for CT-LE. While 3 phases are the minimum required for Siemens' CT-LE analysis application, more phases could theoretically be acquired if further noise reduction is needed.

Expected Benefits of the “Shuttle mode” Approach in CT-LE

- **Temporal and Spatial Accuracy**: The hybrid reconstruction ensures high temporal resolution and consistent CT values, reducing variability in late enhancement imaging.
- **Optimized Noise Reduction**: Averaging multiple phases minimizes noise, providing precise visualization of myocardial enhancement.

Supplementary Figure 3

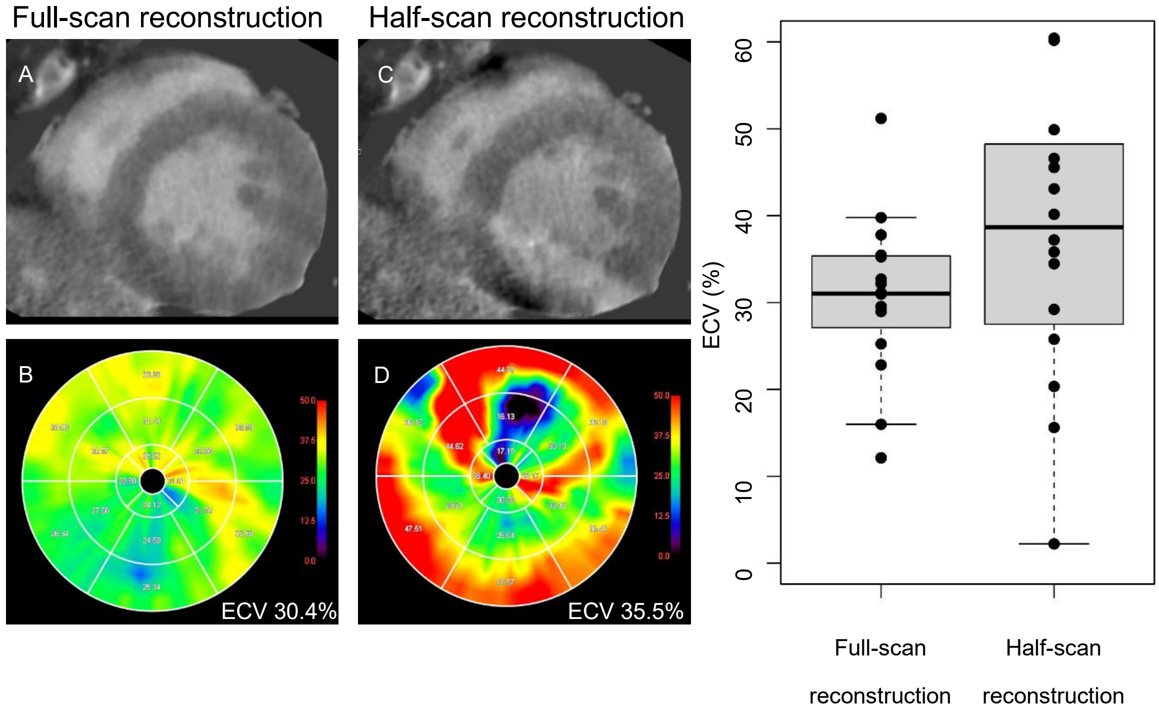

Comparison of extracellular volume (ECV) measurement in dual-energy mode between full-scan reconstruction and half-scan reconstruction in a patient without history of myocardial infarction. A short-axis iodine-specific image (A) and a polar map of ECV (B) derived from full-scan reconstruction and from half-scan reconstruction (C and D). The full-scan reconstruction (A) provides better quality of iodine-specific image than does the half-scan reconstruction (C). Additionally, ECV values calculated from the full-scan images (B) show less variation across segments than those from the half-scan images (D). Box-and-whisker plots showing ECV values of 16 segments in this patient (E) demonstrate that the full-scan reconstruction has less variation in ECV values than does the half-scan reconstruction.

Supplementary Table. Detection of patients with history of MI using CT-LE

	**Dual-energy mode**										**Shuttle mode**	
	Mono-plus 40-KeV		40-KeV		60-KeV		70-KeV		80-KeV			
Observer	A	B	A	B	A	B	A	B	A	B	A	B
Sensitivity	5/8	6/8	4/8	4/8	5/8	6/8	8/8	7/8	8/8	7/8	8/8	8/8
Specificity	7/7	7/7	7/7	6/7	7/7	6/7	7/7	6/7	7/7	6/7	7/7	7/7
PPV	5/5	6/6	4/4	4/5	5/5	6/7	8/8	7/8	8/8	7/8	8/8	8/8
NPV	7/10	7/9	7/11	6/11	6/10	6/8	7/7	6/7	7/7	6/7	7/7	7/7
Accuracy	12/15	13/15	11/15	10/15	12/15	12/15	15/15	13/15	15/15	13/15	15/15	15/15

CT-LE: CT late enhancement, MI: myocardial infarction,
PPV: Positive Predictive Value, NPV: Negative Predictive Value

<70%	70-84%	85-99%	100%
